# The Selenocysteine-Specific Elongation Factor Contains Unique Sequences That Are Required for Both Nuclear Export and Selenocysteine Incorporation

**DOI:** 10.1371/journal.pone.0165642

**Published:** 2016-11-01

**Authors:** Aditi Dubey, Paul R. Copeland

**Affiliations:** Department of Biochemistry and Molecular Biology, Rutgers—Robert Wood Johnson Medical School, Piscataway, NJ, United States of America; University of Toronto, CANADA

## Abstract

Selenocysteine (Sec) is a critical residue in at least 25 human proteins that are essential for antioxidant defense and redox signaling in cells. Sec is inserted into proteins cotranslationally by the recoding of an in-frame UGA termination codon to a Sec codon. In eukaryotes, this recoding event requires several specialized factors, including a dedicated, Sec-specific elongation factor called eEFSec, which binds Sec-tRNA^Sec^ with high specificity and delivers it to the ribosome for selenoprotein production. Unlike most translation factors, including the canonical elongation factor eEF1A, eEFSec readily localizes to the nucleus of mammalian cells and shuttles between the cytoplasmic and nuclear compartments. The functional significance of eEFSec’s nuclear localization has remained unclear. In this study, we have examined the subcellular localization of eEFSec in the context of altered Sec incorporation to demonstrate that reduced selenoprotein production does not correlate with changes in the nuclear localization of eEFSec. In addition, we identify several novel sequences of the protein that are essential for localization as well as Sec insertion activity, and show that eEFSec utilizes CRM1-mediated nuclear export pathway. Our findings argue for two distinct pools of eEFSec in the cell, where the cytoplasmic pool participates in Sec incorporation and the nuclear pool may be involved in an as yet unknown function.

## Introduction

Humans utilize the essential trace element selenium (Se) to make at least 25 essential proteins. These proteins are called selenoproteins and perform myriad functions including the maintenance of cellular oxidative homeostasis, thyroid function, and sperm production [1]. Selenoproteins are produced in both prokaryotes and eukaryotes by the cotranslational insertion of the 21st amino acid selenocysteine (Sec) at specific in-frame UGA codons. Several dedicated factors are required to recode the UGA codon to incorporate Sec instead of recruiting translation termination factors. First is a key feature shared by all selenoprotein mRNAs—a stem-loop structure in the 3’ untranslated region (UTR) called the Sec insertion sequence (SECIS) element. This is the only known essential cis acting element. The remaining are trans acting factors, including the SECIS binding protein 2 (SBP2), the selenocystyl-tRNA^Sec^ (Sec-tRNA^Sec^), and the selenocysteine-specific elongation factor called eEFSec (reviewed in [2]). From several lines of evidence, it is currently believed that a complex containing SBP2 and eEFSec/GTP/Sec-tRNA^Sec^ is assembled on the SECIS element, which allows the ribosomal A-site containing the in-frame UGA codon to be interpreted as Sec. During this process, eEFSec functions as a specialized elongation factor that binds Sec-tRNA^Sec^ with high specificity and delivers it to the ribosomal A-site. The molecular basis for this specificity remains unclear. In addition, since selenoprotein production is differentially regulated under conditions of oxidative stress or limiting selenium supply, it remains unknown whether coordinated regulation of eEFSec and SBP2 occurs.

Several studies have demonstrated that SBP2 and eEFSec form a complex that is presumed to be required for Sec incorporation. In cases where the factors were overexpressed in transfected cells, a stable complex could be isolated by immunoprecipitation [3,4]. With purified factors, however, only a transient complex could be detected that is strictly dependent on the presence of a SECIS element [5]. Further studies examined the subcellular localization of eEFSec and SBP2 under varying conditions to assess the impact of changes in localization of either protein on selenoprotein production. While both eEFSec and SBP2 were shown to shuttle between the nucleus and the cytoplasm, only SBP2 accumulated in the nucleus in response to oxidative stress [6–8]. Additionally, transfected eEFSec and SBP2 were shown to co-localize the nucleus, and both proteins were detected in a co-immunoprecipitation from a nuclear fraction of HEK293 cells in the presence of transfected tRNA^Sec^ [3,9].

Despite these observations, a clear, functional role for nuclear eEFSec and SBP2 has not been demonstrated, though some models have been proposed. The presence of an in-frame UGA codon in selenoprotein mRNAs makes them susceptible to non-sense mediated decay (NMD) under conditions of low selenium (reviewed in [10]). Conditions that increase cellular oxidative stress have been shown to alter selenoprotein production [7,8]. Given that selenoprotein synthesis is sensitive to these changes, the models proposed to explain nuclear localization of Sec incorporation machinery suggest that altered localization is utilized as means to preserve the integrity of Sec insertion components during increased oxidative stress, or to evade NMD in the event of selenium deficiency. However, direct evidence for the functional relevance of nuclear presence eEFSec and SBP2 to efficient selenoprotein production in mammalian cells has not been shown.

In this study, we examined the subcellular distribution of eEFSec in the context of altered selenoprotein production to understand the functional significance of its nucleocytoplasmic shuttling. We identified several novel sequences that are critical for both the activity and shuttling of eEFSec, and we demonstrate that disrupting eEFSec localization does not alter selenoprotein production in rat hepatoma cells. In addition, we utilized a newly constructed SBP2-null cell line and demonstrated that eEFSec localization is unaffected in the absence of SBP2, despite dramatic differences in selenoprotein production. Taken together, our results indicate that the role of nuclear eEFSec is not linked to its role in Sec incorporation, but that functionally important sequences in the protein are required for proper nuclear localization.

## Materials and Methods

### Constructs

FLAG-eEFSec was PCR amplified from the coding region of mouse eEFSec as described previously [11]. The resulting product was cloned into pcDNA3.1 for expression in mammalian cells using the TOPO-TA cloning kit (Invitrogen). Penta-alanine mutants of eEFSec Domains I, II and III were generated using the QuikChange Site-Directed Mutagenesis Kit (Agilent) as per manufacturer’s instructions. Mutations were confirmed by DNA sequence analysis. eEFSec Domain IV mutants were created as described previously [11].

### Protein expression and purification

All recombinant proteins used in this study were FLAG-tagged and purified from E. coli BL21 as described previously [11]. Purified proteins were analyzed for purity using SDS-PAGE, followed by Coomassie blue staining and quantitation against an ovalbumin standard using Carestream molecular imaging software.

Cell culture and transfection—All cell lines were maintained at 37°C with a humidified 5% CO2 atmosphere. McArdle cells (ATCC) were cultured in HyClone DMEM with F-12 (GE Lifesciences) containing 10% FBS. Transfections were performed in McArdle and HAP1 cells using jetPRIME (Polyplus) according to the manufacturer's protocol. Cells were seeded 24 hours prior to transfection at a density of 3–4 ×10^5^ per well in a 6-well plate. Cells were given fresh media after 24 hours and collected 48 hours post-transfection in NP40 lysis buffer (50 mM Tris pH 8.0, 150 mM NaCl, 1% NP-40, 1 mM dithiothreitol (DTT) and 0.5 mM phenylmethylsulfonyl fluoride [PMSF]). Stable cell lines were generated by using G418 to select for cells expressing the G418 resistance gene present in pcDNA3.1. McArdle cells stably expressing with pcDNA3.1 expressing FLAG-tagged proteins were grown in 750–800 μg/mL of G418 (Gibco). HAP1 cells (Horizon Genomics) were grown in Iscove's Modified Eagle Medium (IMEM; Gibco) with 10% FBS and were transfected as described above.

### In Vitro Translation and Sec Incorporation Assay

Sec incorporation activity was assessed using a luciferase reporter based in vitro translation assay described in prior work [11,12]. Briefly, luciferase mRNA containing a UGA/Sec codon at position 258 and the Sepp1 3’UTR was used to measure Sec insertion at UGA in a SECIS-dependent manner. To measure non-specific readthrough at the UGA codon, the luciferase mRNA was modified with both the SECIS elements deleted in the 3’ UTR using QuikChange. In 12.5 μL reaction volume, 125 ng of these reporter mRNAs were added to 6.5 μL of wheat germ extract (Promega), 2 μM SBP2, 4 μM eEFSec (wild-type or mutant), 1.25 ug of total testis tRNA, 250 mM KOAc, and 250 mM of amino acid mix (supplied with the Wheat Germ Extract; Promega). Reactions were incubated at 24°C for 2 hours and luminescence was measured in a 96-well plate luminometer (Berthold TriStar). Sec incorporation activity was normalized to the non-specific readthrough and reported as relative luminescence units (RLU).

Antibodies—The following antibodies were used in the western blot and immunofluorescence analyses: Mouse anti-FLAG (Sigma, F1804), mouse anti-GAPDH (Millipore MAB374), rabbit anti-SBP2 (Proteintech, 12798-1-AP), rabbit anti-GPX1 (Abcam, ab22604) rabbit anti-HDAC1 (Thermo Scientific, PA1-860), mouse anti-Cyclin B1 (Santa Cruz, sc-245). All HRP and Cy3 conjugated secondary antibodies were obtained from Invitrogen.

### Immunofluorescence and Confocal Microscopy

Cells were seeded on coverslips coated with poly-L-lysine (Sigma) and immunofluorescence was performed 48 hours after transfection for transient transfection, or 2–3 weeks after antibiotic selection for stable cell lines. All steps were performed on a rotating platform, unless otherwise noted. Coverslips were fixed in 4% paraformaldehyde at pH 7.4 (Sigma, 441244) for 10 min at room temperature. Cells were washed three times with 1X PBS and blocked at room temp for 1 hr in blocking solution #1 (10%FBS, 0.2% Triton X-100 in PBS). Anti-FLAG antibody (1:3000) was added for overnight incubation with rotation at 4°C. Cells were washed four times with 1X PBS and incubated with 1:2500 Cy3 conjugated mouse IgG in blocking solution #2 (10% FBS in PBS) for 2 hours at room temp. A 1:1000 dilution in 1x PBS of 300 μM 4′,6-Diamidino-2-phenylindole dihydrochloride (DAPI; Sigma D9542) was used for 10 mins to stain nuclei. Coverslips were mounted on slides using Fluoromount-G (Affymetrix, 00-4958-02) and imaged using a spinning disc confocal microscope (Upright Zeiss AxioImager Z1; RWJMS Imaging Core Facility). All images were captured at 63x magnification, unless otherwise stated. Image analysis was done using the FIJI open source software [13]. For the leptomycin B (LMB) experiments, McArdle cells were exposed to 5, 10 and 20 nM LMB (Sigma, L2913) from 2 to 22 hours to determine the optimal dose without cytotoxicity. 20 nM LMB was well tolerated up to 8 hours but we chose a shorter incubation of 2 hours to avoid side-effects as LMB has been shown to reversibly induce cell cycle arrest in G1 and G2 phases of eukaryotic cell cycle [14,15]. Nuclear/Cytoplasmic signal ratios were calculated using Fiji. The mean fluorescence intensity of the nucleus was compared to that of the cytoplasm to generate a nuclear/cytoplasmic signal ratio. All cells in the plane of the image with non-overlapping nuclei were measured and the average nuclear/cytoplasmic ratio was calculated. Nuclei were demarked by DAPI staining in merged images. If the ratio of nuclear/cytoplasmic signal was 1 or greater, the cell was considered positive for nuclear localization. Ratios of less than 1 were classified as nuclear exclusion. P-values were obtained by using a two-tailed t-test with unequal variance, and p-values < 0.05 were considered significant.

### ^75^Se labeling

24 hours prior to harvesting, cells were given fresh media containing 100 nM of ^75^Se (specific activity 570 Ci/g; Hartmann Analytics, Braunschweig, Germany). On the next day, cells were washed once with cold PBS and lysed in NP-40 buffer. Extracts were cleared by centrifugation at 17,000 x g for 10 min at 4°C and protein concentration was measured using BCA protein assay (Pierce). Equal amounts of protein were resolved by 15% SDS-PAGE and visualized by phosphorImaging (GE Healthcare) or by 12% SDS-PAGE for western blot analysis.

## Results

### Expression of recombinant eEFSec in mammalian cells

**Fig 1:**
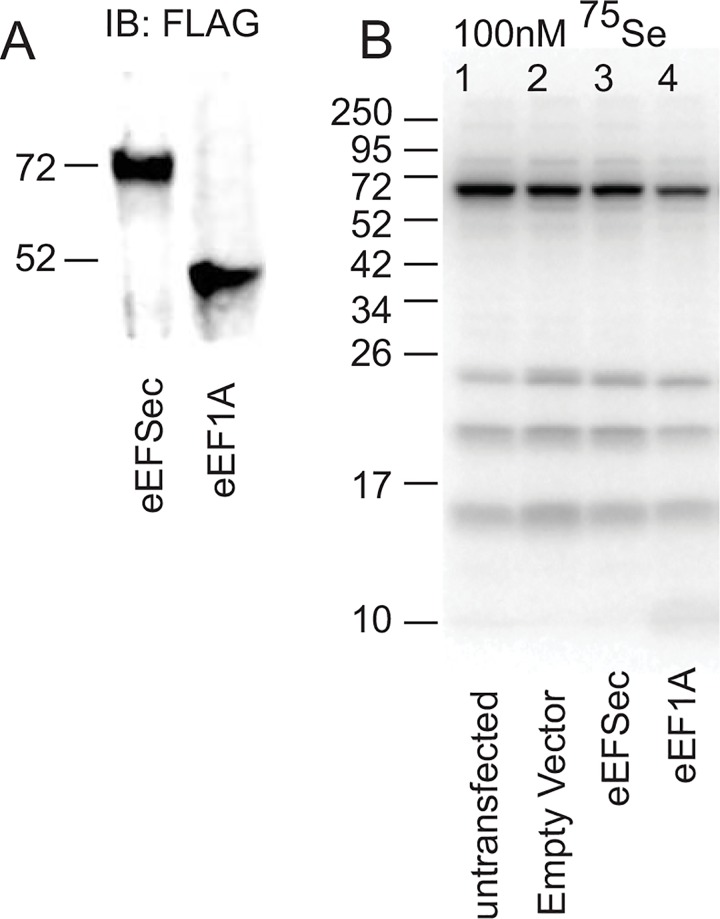
Expression of recombinant eEFSec and eEF1A in McArdle cells. (A) Immunoblot analysis of protein lysates from FLAG-eEFSec and FLAG-eEF1A transfected cells probed with anti-FLAG antibody. (B) Metabolic labeling of untransfected (lane 1), empty vector control (lane 2), FLAG-eEFSec (lane 3) and eEF1A (lane 4) expressing McArdle stable cell lines using 100 nM ^75^Se. Lysates were resolved by 15% SDS-PAGE 24 hours after the addition of label and analyzed by autoradiography to detect endogenous selenoprotein expression.

In an effort to gain more insight into a function for eEFSec nuclear localization, we analyzed the subcellular localization of N-terminally FLAG-tagged mouse eEFSec that was stably transfected into a rat hepatoma cell line, McArdle 7777. McArdle cells express relatively high levels of SBP2 [16], and have been shown to exhibit dramatic changes in selenoprotein production as a function of added selenium [9]. Additionally, McArdle cells are easy to transfect and have proportional nuclear and cytoplasmic compartments, making them suitable for our study. Fig 1A shows immunoblot blot analysis of McArdle cells stably expressing FLAG-eEFSec or a FLAG-tagged version of mouse eEF1A as a control, and both proteins are easily detectable at the expected molecular masses. Importantly, this is the same version of eEFSec that we have previously shown to be highly active in an eEFSec-dependent Sec incorporation assay [12]. To evaluate the impact of transfected FLAG-eEFSec on endogenous selenoprotein production in McArdle cells, metabolic labeling with ^75^Se-selenite was performed. The stably transfected McArdle cells were exposed to 100 nM ^75^Se-selenite for 24 hours and radiolabeled selenoproteins were resolved by SDS-PAGE and detected by digital autoradiography (Fig 1B). This experiment shows that overexpressing eEFSec in McArdle cells does not have a significant positive or negative effect on selenoprotein production when compared to the eEF1A control, suggesting that the endogenous protein is not limiting or that selenoprotein mRNAs are not accessible by the transfected pool of eEFSec. Note that our attempt to reduce or eliminate endogenous eEFSec in McArdle cells was not successful, so all of the data presented here is in the context of wild-type endogenous eEFSec.

### Nuclear localization signals are present in Domain IV of eEFSec

**Fig 2:**
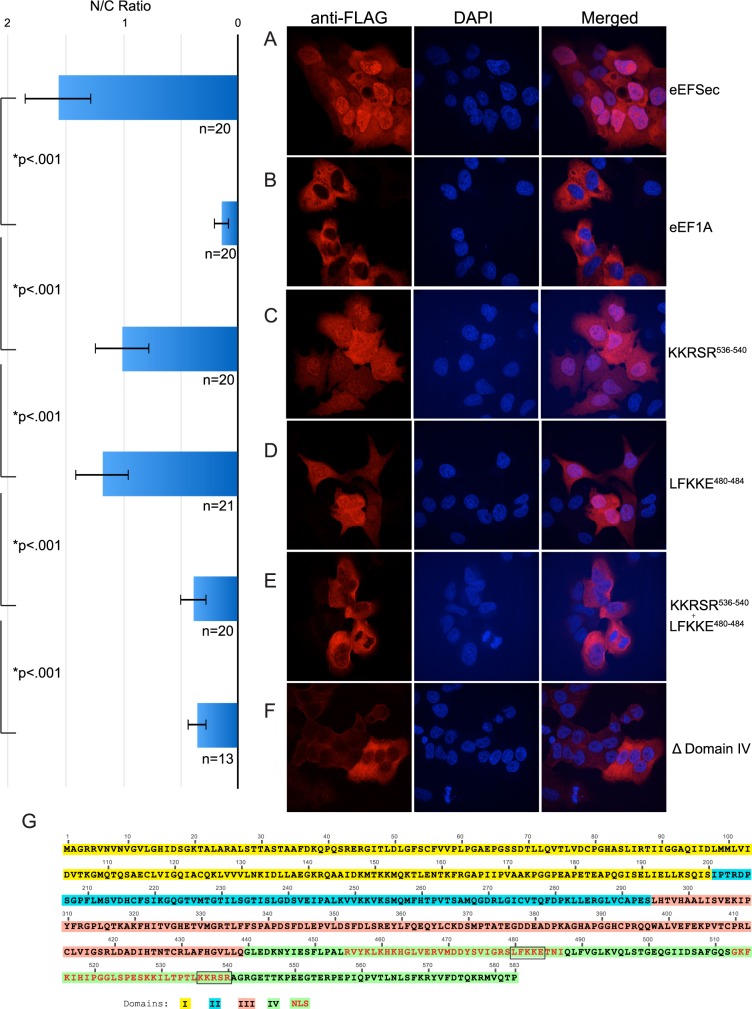
eEFSec is a nucleocytoplasmic shuttling protein with a bipartite NLS. Immunofluorescence of McArdle cells stably expressing (A) FLAG-eEFSec, (B) FLAG-eEF1A, (C) FLAG-eEFSec KKRSR^536-540^ (D) FLAG-eEFSec LFKKE^480-484^ (E) double mutant FLAG-eEFSec LFKKE^480-484^ + KKRSR^536-540^ (F) FLAG-eEFSec Domain IV deletion. Anti-FLAG was detected by Cy3 conjugated secondary antibody and is indicated in red (left panels). The nuclear stain DAPI is in blue (middle panels). A merged image of the two channels (right panels) shows eEFSec co-localizes with DAPI while eEF1A does not. All images taken at 63x magnification using a confocal microscope. The nuclear/cytoplasmic (N/C) ratios for the images were determined as described in the Methods section and are shown graphically with standard deviation, p values and values for n. G) eEFSec domains are color-coded as shown and the sequences predicted as nuclear localization sequences by NLSMapper are highlighted in red. The two sequences mutated to penta-alanine are boxed.

Previous work showed that transfected eEFSec was localized to the nucleus in HEK293 cells [6]. That study used the PSORTII algorithm, which identified the sequence PTLKKRSR in Domain IV to be important for its nuclear localization, and mutation of the two lysine residues to glutamic acid was sufficient to result in nuclear exclusion. Subcellular distribution of eEFSec also appeared to differ in a cell-type specific manner [6]. To investigate eEFSec localization in McArdle cells, immunofluorescence derived from anti-FLAG immunohistochemistry was performed on McArdle cells that were stably transfected with FLAG-eEFSec. As a control we also stably transfected FLAG-tagged elongation factor 1A (FLAG-eEF1A), the canonical translation elongation factor that does not play a role in Sec incorporation. Confocal microscopy was used to examine the subcellular location of FLAG signal with respect the nuclear stain 4’,6-Diamidino-2-Phenylindole (DAPI). Overlapping DAPI and FLAG signal with a nuclear/cytoplasmic signal ratio of less than 1 was interpreted as nuclear localization as descried in the materials and methods, whereas mutually exclusive FLAG and DAPI signals were indicative of nuclear exclusion. Our results show that eEFSec consistently localized to both the cytoplasm and the nucleus, whereas eEF1A was always exclusively found in the cytoplasm (Fig 2A and 2B).

eEFSec is comprised of three canonical elongation factor domains followed by a unique fourth domain (Domain IV) that is required for Sec incorporation [11,17]. In an analysis of conserved regions in Domain IV, several discrete sequences were found to be crucial for Sec incorporation activity. In that study it was found that mutating the sequence KKRSR^536-540^ to alanine residues (referred to as a “penta-alanine” mutation) rendered eEFSec inactive and unable to bind SBP2 or Sec-tRNA^Sec^ [11]. In order to test for defects in nuclear localization resulting from this mutation, we stably transfected McArdle cells with FLAG-eEFSec harboring the KKRSR^536-540^ penta-alanine mutation. Surprisingly, our analysis did not reveal any nuclear exclusion of eEFSec containing the KKRSR^536-540^ mutation (Fig 2C). To find other residues that may contribute to eEFSec nuclear localization, we employed the NLSMapper tool [18]. NLSMapper identifies all four classes of classical NLSs recognized by importin-α, which is the nuclear import receptor for a majority of proteins, and recognizes both monopartite and bipartite signals [18,19]. Interestingly, NLSMapper revealed that eEFSec may contain a bipartite NLS sequence in Domain IV with a 25 amino acid linker (Fig 2G). The stretches of sequences highlighted in red were scored 5.7 (residues 455 to 487) and 5.9 (residues 513 to 540) respectively, indicating that eEFSec is predicted by NLSMapper to be a nucleocytoplasmic shuttling protein. In order to test the hypothesis that eEFSec contains a bipartite NLS, we tested a penta-alanine mutation at LFKKE^480-484^, which is predicted to be at the C-terminus of the first NLS sequence. To that end we stably transfected FLAG-eEFSec LFKKE^480-484^ and a double penta-alanine mutant at KKRSR^536-540^ and LFKKE^480-484^ and performed anti-FLAG immunoflourescence. While the LFKKE^480-484^ mutation in isolation did not show a strong localization defect (Fig 2D), the double mutant protein was excluded from the nucleus (Fig 2E). Consistent with the idea that domain IV harbors the complete NLS, the deletion of Domain IV resulted in nuclear exclusion (Fig 2F). Overall, these results indicate that eEFSec is present in both the nuclear and cytoplasmic compartment of McArdle cells, and contains a bipartite NLS sequence in the domain of eEFSec that is predicted to be flexible and prone to conformational changes. These results largely coincide with prior work, but the lack of eEFSec nuclear exclusion upon mutation of KKRSR residues does conflict with the previous data [6]. In that study, it is possible that the charge reversal resulting from their use of a KK536/537EE mutation may have had a dominant negative effect on nuclear localization by affecting global domain structure. Importantly, however, we have here shown that two different versions of eEFSec that were previously shown to be completely inactive for Sec incorporation (KKRSR^536-540^ and LFKKE^480-484^) still show nuclear localization, thus providing the first evidence that Sec incorporation activity and nuclear localization are not necessarily linked.

### Conserved residues in eEFSec are important for nucleo-cytoplasmic shuttling

**Fig 3:**
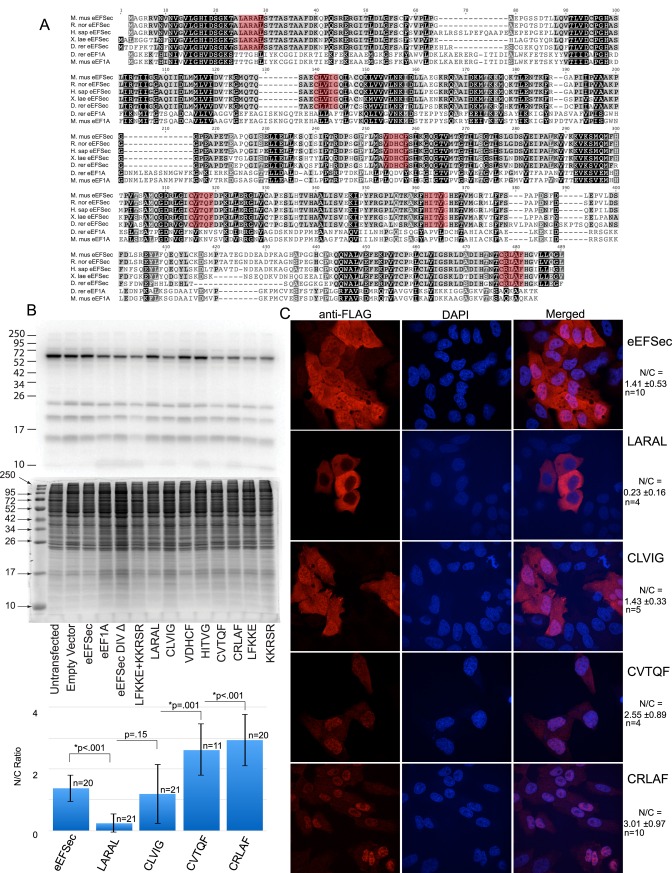
Mutagenesis of conserved sequences unique to eEFSec result in localization defects. (A) ClustalW alignment of vertebrate eEFSec sequences from domains I, II and III and eEF1A. Highly conserved residues are highlighted in black, semi-conserved residues highlighted with grey and non-conserved are unshaded. Red boxes enclose residues in eEFSec selected for penta-alanine mutagenesis. (B) Autoradiography analysis of cells expressing penta-alanine mutants labeled with 100 nM ^75^Se for 24 hours, lysed and resolved on 15% SDS-PAGE (first four lanes are a duplicate of that shown in Fig 1B). The same gel stained with Coomassie Blue is shown below. (C) Immunofluorescence analysis of stable cell lines generated from penta-alanine mutants in eEFSec domains I, II and III. The nuclear/cytoplasmic (N/C) ratios for the images were determined as described in the Methods section and are shown graphically with standard deviation, p values and values for n.

While NLS sequences are easily categorized into different classes, NES sequences are diverse and typically contain regularly spaced hydrophobic residues. Therefore, instead of using bioinformatic approaches to attempt to predict eEFSec NES, we analyzed a subset conserved sequences in eEFSec that are absent in eEF1A. Since eEF1A is excluded from the nucleus, this approach will allow for identification of sequences unique to eEFSec that are required for nuclear export. Sequences from vertebrate species corresponding to eEFSec domains I, II and III were aligned and compared with conserved regions in eEF1A (Fig 3A). These “non-eEF1A” sequences were used to generate a series of penta-alanine mutants to identify key residues involved in eEFSec nuclear export (Fig 3A; mutated residues are in red boxed regions). All selected regions contained at least two hydrophobic residues, variously spaced. The selection of sequences was not comprehensive but rather evenly distributed across the domains. The penta-alanine mutant versions of FLAG-eEFSec were stably transfected into McArdle cells, which were then metabolically labeled with ^75^Se to check for any significant, dominant impact of the mutations on selenoprotein synthesis. As seen in Fig 3B, although some minor differences were observed, the overall profile of selenoprotein expression was maintained, indicating that the mutations do not have a dominant-negative effect on eEFSec activity. In general we observe a fairly high degree of variability in the metabolic labeling of these cells, perhaps due to their tendency to clump, so we can only make a qualitative assessment that the expression of eEFSec mutant proteins did not drastically change selenoprotein expression.

Stably transfected McArdle cells expressing all six eEFSec mutants were analyzed as before and compared to wild-type eEFSec and eEF1A. As shown in Fig 3C, the mutants showed varying localization defects. In the case where the sequence CLVIG^116-120^ was mutated to alanine residues (denoted as CLVIG^116-120^) there were no significant changes, and the protein localized similarly to wild-type eEFSec. However, mutation of LARAL^23-27^ in Domain I, which resembles a known NES most closely, did not result in nuclear accumulation, rather this sequence appears to have NLS function since the protein localized exclusively to the cytoplasm (Fig 3C). Intriguingly, these residues are located close to the switch I region of the GTPase domain in eEFSec [17]. Similarly, the eEFSec Domain I residues IIDLMMLVI^95-103^ that were previously reported as the putative NES [6] are located adjacent to eEFSec switch II region [17]. These were not included in our analysis due to many of these residues also being conserved in eEF1A. However, deletion of these residues resulted in almost complete exclusion of eEFSec from the nucleus [6]. Taken together with our observations, these data may suggest that normal GTPase activity of eEFSec is critical for nuclear import. Interestingly, the CVTQF^275-279^ mutation in Domain II and CRLAF^429-433^ mutation in Domain III led to reduced expression and predominantly nuclear accumulation of eEFSec (Fig 3C). These data suggest that several conserved sequences in Domains I, II and III are important for nucleocytoplasmic shuttling of eEFSec. Another possibility for these results is that the conserved residues are also important for appropriate folding and function of eEFSec, in absence of which the protein fails acquire a favorable conformation for nucleocytoplasmic shuttling. Further in-depth analysis of the localization signals will be required to distinguish these possibilities.

**Fig 4:**
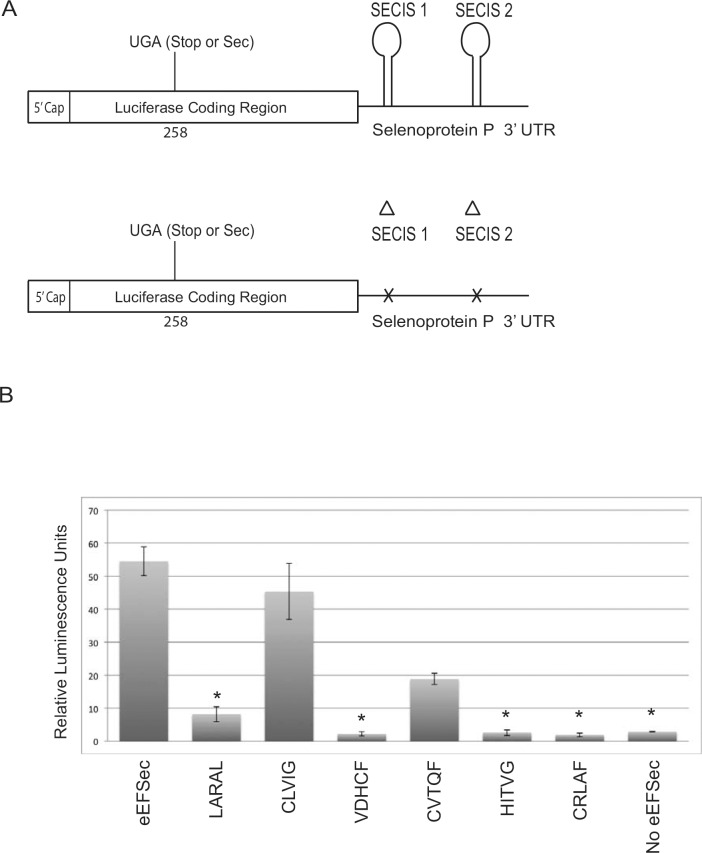
Penta-alanine mutants show a range of Sec incorporation activity. (A) Diagram of the luciferase reporter constructs used in Sec incorporation assay. Luciferase coding region with a UGA at position 258 and SelP 3’ UTR serves as a measure of Sec incorporation, whereas the same reported with both the SECIS elements deleted from SelP 3’ UTR serves as a measure of non-specific readthrough. (B) Sec incorporation activity of 4uM purified wild-type FLAG-eEFSec and the mutant proteins as indicated added to a wheat germ lysate in vitro translation reaction (see Methods section for details). The data represents the ratio of wild-type versus SECIS mutant luciferase activity. The data is derived from three independent experiments and standard deviations are shown. P-values were obtained by using a two-tailed t-test with unequal variance, and p-values < 0.05 were considered significant as indicated by an asterisk.

To investigate whether eEFSec mutants generated in the previous experiment are functional for Sec incorporation and also to determine whether nucleocytoplasmic shuttling of eEFSec correlates with selenoprotein synthesis, we examined whether the shuttling and Sec incorporation capabilities of eEFSec are coupled. Ideally this would be tested in cells lacking endogenous eEFSec, but such cells were not recovered from a genome scale CRISPR-Cas9 screen, indirectly indicating that the deletion of eEFSec is lethal [20]. Therefore, we tested each of the penta-alanine mutant proteins in a wheat germ based in vitro Sec incorporation assay [12]. This assay is dependent on the exogenous addition of each factor known to be involved in Sec incorporation, and has been demonstrated to be eEFSec-dependent [11,21]. Reporter mRNAs used in this study consisted of a modified luciferase coding region with an in-frame UGA codon followed by the selenoprotein P (Sel P) 3’ untranslated region (UTR) with either wild-type SECIS elements to measure Sec incorporation or deleted SECIS elements to measure Sec-independent, non-specific readthrough (Fig 4A). The Domain I—III mutant proteins were purified from bacteria and tested in equal concentrations in wheat germ extract. Sec incorporation activities of these eEFSec mutants spanned a broad range (Fig 4B). In Domain I, while the CLVIG^116-120^ mutation showed Sec incorporation activity that was only slightly reduced as compared to wild-type, LARAL^23-27^ showed highly reduced activity. Domain II mutant VDHCF^214-218^ was inactive, with luminescence readings almost the same as those observed without eEFSec. However, partial activity was observed with Domain II mutant CVTQF^275-279^. Both mutants of Domain III, HITVG^322-326^ and CRLAF^429-433^, were inactive as well. These results indicate that residues in Domains I, II and III, that are important for nucleocytoplasmic shuttling of eEFSec are also important for function, especially since mutation of CLVIG^116-120^ residues disrupts neither activity nor localization. Based on the combined localization and Sec incorporation activity results, there is no significant correlation between Sec incorporation activity of eEFSec and its shuttling between the nucleus and the cytoplasm. From these data, we can conclude that the role of nuclear eEFSec is likely unrelated to its direct role in Sec incorporation.

### Localization of eEFSec is independent of selenium levels

**Fig 5:**
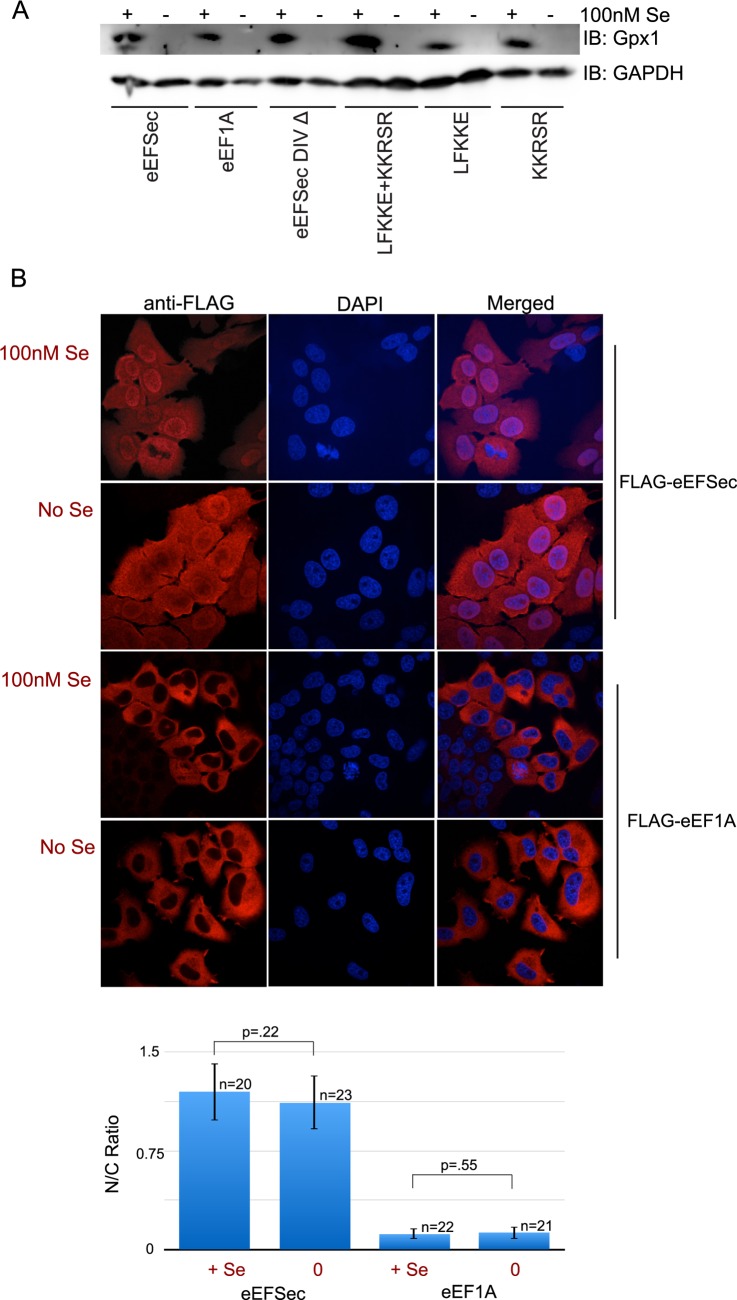
eEFSec localization is unchanged during altered selenoprotein production. McArdle cells stably expressing FLAG-eEFSec were exposed to 100nM selenium or left unsupplemented as indicated. (A) Immunoblot analysis of protein lysates from McArdle cells stably expressing eEFSec, eEF1A, the mutants LFKKE^480-484^, KKRSR^536-540^ and double mutant FLAG-eEFSec mLFKKE^480-484^ + KKRSR^536-540^ were sequentially probed with anti-GPX1 (top row), and anti-GAPDH (bottom row) antibodies. (B) Immunofluorescence of McArdle cells stably expressing FLAG-eEFSec (top two rows) or FLAG- eEF1A (bottom two rows) exposed to 100nM Se or unsupplemented as indicated. The nuclear/cytoplasmic (N/C) ratios for the images were determined as described in the Methods section and are shown graphically with standard deviation, p values and values for n.

In order to further probe the relationship between Sec incorporation and the nuclear localization of eEFSec, we investigated the effect of selenium supplementation on subcellular localization of eEFSec. In McArdle cells, the expression and activity of glutathione peroxidase 1 (GPX1) decreases dramatically in conditions of depleted selenium, making it a useful indicator of cellular selenium status [22]. Therefore, we utilized lack of GPX1 expression as an indicator of reduced selenoprotein production in McArdle cells and examined eEFSec distribution under these conditions. Western blot analysis of McArdle cells stably expressing wild-type or mutant FLAG-eEFSec or the FLAG-eEF1A control showed no detectable GPX1 protein in the absence of supplemental selenium (Fig 5A). The analysis of subcellular localization under these conditions did not show any change as a result of selenium status (Fig 5B). As expected, no changes were seen in FLAG-eEF1A localization. Overall, these findings further demonstrate that the nuclear localization of eEFSec does not change even when the cell undergoes significant changes in selenoprotein production.

### eEFSec shuttling is blocked by the CRM1 inhibitor Leptomycin B

**Fig 6:**
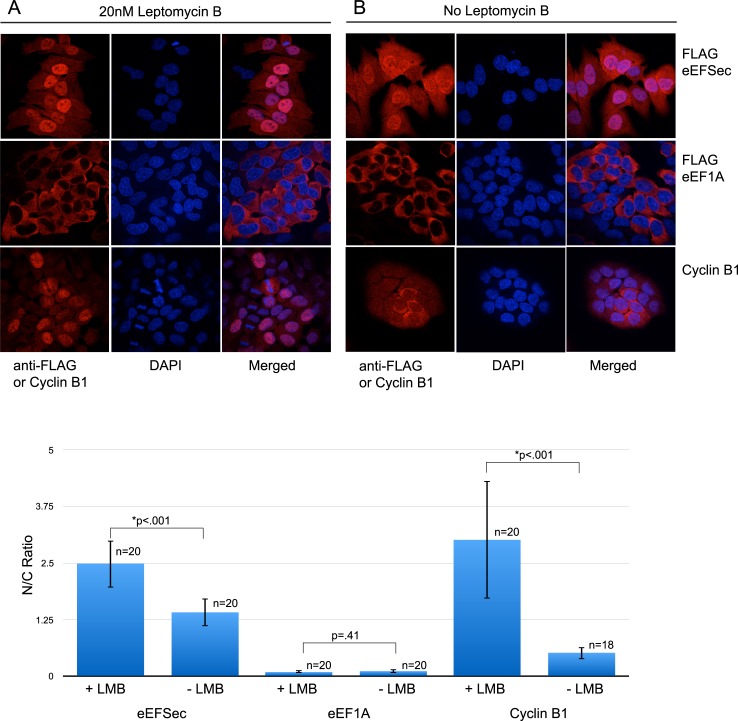
Increased retention of eEFSec in the nucleus upon Leptomycin B treatment. (A) McArdle cells stably expressing FLAG-eEFSec or FLAG- eEF1A were exposed to 20nM LMB (A) or equivalent volume of water (B) for 2 hours, then analyzed for subcellular localization via immunofluorescence. Anti-FLAG immunoflourescence was analyzed for McArdle cells stably expressing eEFSec or eEF1A as indicated. Anti-Cyclin B1 immunofluorescence was analyzed in FLAG-eEFSec expressing McArdle cells. The nuclear/cytoplasmic (N/C) ratios for the images were determined as described in the Methods section and are shown graphically with standard deviation, p values and values for n.

Having established that eEFSec utilizes a canonical bipartite NLS for nuclear import, we next set out to determine the pathway for nuclear export. A major export receptor for the bulk of nuclear export in mammalian cells is CRM1. It has a broad substrate range and mainly interacts with leucine-rich nuclear export signals (NES), but proteins with other hydrophobic residues substituted for leucine (such as isoleucine, valine, phenylalanine or methionine) have also been found to be CRM1 targets [23]. In an effort to determine whether eEFSec utilizes the CRM1 pathway for nuclear export, we examined the effect of the CRM1 inhibitor leptomycin B (LMB) on the subcellular localization of eEFSec. McArdle cells stably expressing FLAG-eEFSec and FLAG-eEF1A were exposed to LMB for 2 hours, after which their subcellular localization was determined by immunofluorescence. In the presence of LMB, eEFSec becomes more concentrated in the nucleus, demonstrating that it contains one or more CRM1 responsive NES sequences whereas eEF1A remains unchanged (Fig 6A). As a positive control, the localization of Cyclin B1 was assessed under similar conditions. Cyclin B1 is a cell-cycle protein that it is known to have a leucine-rich, LMB-responsive NES [15]. Cyclin B1 readily translocates to the nucleus upon LMB treatment, resulting in predominantly nuclear localization. In the absence of LMB, Cyclin B1 is visualized as diffuse cytoplasmic stain, and eEFSec is present in both the nucleus and cytoplasm in relatively similar amounts (Fig 6B). Finally, to rule out any impact of cell-cycle progression on translocation of eEFSec, McArdle cells were synchronized using serum deprivation for 24 hours prior to localization analysis. No significant changes were observed in eEFSec subcellular localization at different time points during the cell cycle (data not shown). Collectively, these data indicate that eEFSec is likely exported from the nucleus by CRM1 through NESs containing hydrophobic residues.

### eEFSec localization is unchanged in the absence of SBP2

**Fig 7:**
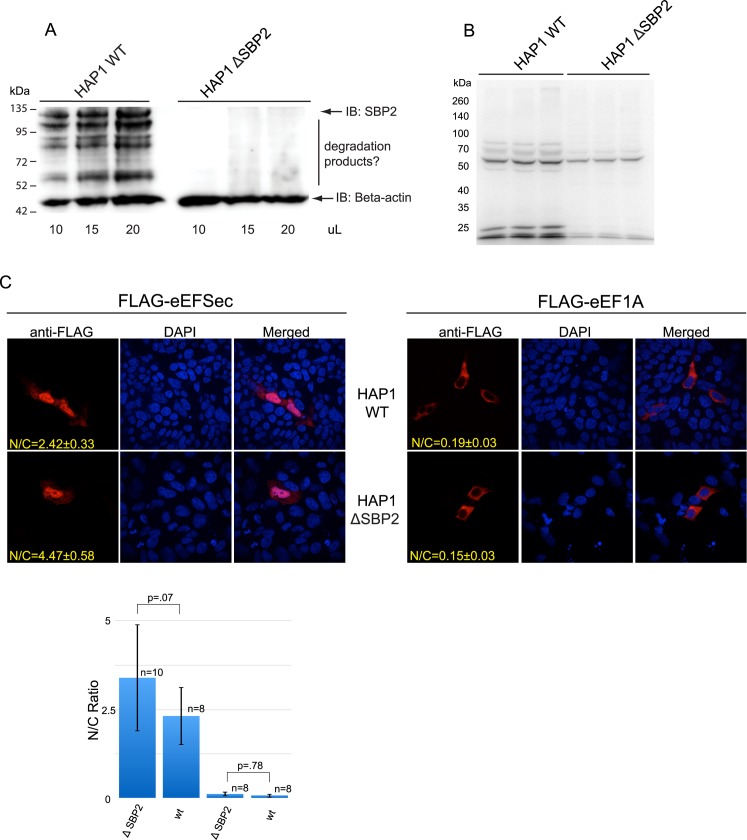
Eliminating SBP2 expression does not alter eEFSec localization. (A) Wild-type and SBP2 null HAP1 cells were lysed and the indicated volumes were resolved on 8% SDS-PAGE for immunoblotting and probed with 1:1000 anti-SBP2 antibody. The majority of SBP2 is detected between 135 and 95 kD and several presumed degradation products are also seen at lower molecular weights. The membrane was washed and probed for β-actin as a loading control. (B) Wild-type and SBP2-null HAP1 cells were labeled with 100 nM ^75^Se for 24 hours, lysed and resolved on 8% SDS-PAGE for autoradiography analysis. Samples were loaded in triplicate. (C) Wild-type and SBP2-null HAP1 cells were transiently transfected with FLAG-eEFSec and FLAG-eEF1A and analyzed via anti-FLAG immunofluorescence 48 hours after transfection. The nuclear/cytoplasmic (N/C) ratios for the images were determined as described in the Methods section and are shown graphically with standard deviation, p values and values for n.

Previous reports have suggested that eEFSec and SBP2 translocate to the nucleus and form an mRNP complex to facilitate Sec incorporation [6,8]. To test whether SBP2 expression is important for nucleocytoplasmic shuttling of eEFSec, we analyzed its localization in the absence of SBP2. To that end, we obtained an SBP2 null HAP1 cell line harboring a 163 base pair insertion immediately following the codon for Gly 583 derived from a CRISPR guide RNA sequence targeting exon 13 (Horizon Genomics). This created a frameshift that prevents the translation of the essential RNA binding domain within SBP2. Genomic sequencing confirmed the insertion and as shown in Fig 7A, we are unable to detect SBP2 protein in lysates derived from the SBP2-null HAP1. The multiple bands observed in the wild-type HAP1 cell line probably represent SBP2 degradation products since the pattern changes as a function of cell density (data not shown). Based on these data, however, we cannot exclude the possibility that multiple isoforms of SBP2 are expressed in this cell type. Metabolic labeling with ^75^Se showed significant reduction in selenoprotein production in SBP2-null HAP1 cells as compared to wild-type HAP1 (Fig 7B). The fact that selenoprotein production was not eliminated in these cells is consistent with similar data obtained from liver-specific SBP2 knockout mice [24].

Subsequently, these cells were transiently transfected with FLAG-eEFSec and FLAG-eEF1A, and their subcellular localization was analyzed as before. Although transfection efficiency was low, FLAG-eEFSec was detected in both the nucleus and cytoplasm in both wild-type and SBP2-null HAP1 cells, and eEF1A was excluded from the nucleus in both cases (Fig 7C). These results demonstrate that SBP2 expression does not alter subcellular distribution of eEFSec, suggesting that SBP2 does not play a role in eEFSec nuclear localization.

## Discussion

Prior work has suggested that the Sec incorporation machinery responds to conditions of oxidative stress or lack of selenium by altering its subcellular localization and in doing so, escapes unfavorable conditions and maintains its integrity. eEFSec has been readily detected in the nucleus regardless of changes in oxidative stress and selenium status, whereas only low levels of SBP2 can be detected in the nucleus under oxidative stress [7,8]. There is also some evidence to suggest that nuclear eEFSec exists as a higher molecular weight complex detectable by a non-denaturing gel electrophoresis [6]. In addition, immunoprecipitation experiments in HEK293 cells transfected with Sec incorporation factors showed that in the presence of excess tRNA^Sec^, a complex between eEFSec and SBP2 could be detected in both the nucleus and the cytoplasm [3]. Based on these observations, it was suggested that a complex between SBP2/eEFSec/Sec-tRNA^Sec^ on the SECIS element of a selenoprotein mRNA is assembled in the nucleus and exported to the ribosomes near the nucleus-ER interface in order to escape NMD and allow for efficient translation of selenoproteins [3,6]. It was also proposed as a means for the Sec incorporation machinery to evade cytosolic oxidative stress [7]. However, there are conflicting reports on changes in selenoprotein production during oxidative stress [7,8]. While SBP2 readily localizes to the nucleus in response to H_2_O_2_-induced oxidative stress, whether or not selenoprotein synthesis is affected depends on the levels of oxidative stress. During low levels of oxidative stress of short duration, selenoprotein production is increased [8], whereas reduced levels of synthesis are observed after high or prolonged levels of oxidative stress [7]. Given these observations, a direct relationship between nuclear presence of eEFSec and SBP2 and efficient selenoprotein translation has been difficult to establish.

In this work we attempted clarify some of these findings and determine a role for nuclear eEFSec by assessing its localization in the context of fundamentally altered Sec incorporation either by selenium deprivation or SBP2 deletion. Consistent with previous reports, our results show that eEFSec is readily detectable in the nucleus of McArdle7777 rat hepatoma cells, whereas the canonical translation elongation factor eEF1A is found only in the cytoplasm [6,8,25]. McArdle cells display altered selenoprotein production in conditions of low selenium, as evidenced by lack of GPX1 expression. Selenium regulates production of selenoproteins to varying degrees, and GPX1 mRNA is especially sensitive to selenium levels, showing up to 90% reduction in mRNA abundance under selenium deficient conditions (reviewed in [26]). Using this as reliable marker for cellular selenium status, we show that subcellular distribution of eEFSec is unchanged under conditions that induce changes in selenoprotein production. These results are consistent with a previous report in which eEFSec nuclear localization in HEK293 cells was shown to be unchanged between conditions of unsupplemented and supplemented selenium [8]. In this study however, conditions of unsupplemented selenium did not show any demonstrable decrease in GPX1 levels or activity, suggesting that a truly selenium-deficient condition was not achieved. Our results establish that eEFSec nuclear localization is independent of cellular selenoprotein production, suggesting that nuclear localization of eEFSec is not coupled to regulated selenoprotein synthesis. In addition, since the concentration of Sec-tRNA^Sec^ is directly related to the selenium concentration, the work shown here excludes the possibility that eEFSec nuclear localization is regulated by an association with Sec-tRNA^Sec^ in the nucleus.

SBP2 levels do not impact eEFSec distribution—To further investigate the link between eEFSec nuclear localization and selenoprotein synthesis, we obtained an SBP2 null cell line from Horizon Genomics. This, therefore is the first homogeneous cell population that lacks SBP2 expression as confirmed by genomic sequencing and immunoblot analysis. The fact that substantial selenoprotein synthesis remains is similar to the results from a liver-specific SBP2 knockout in the mouse [27], which may implicate a role for the SBP2 orthologue, SECISBP2L, in promoting Sec incorporation when SBP2 is limiting. Interestingly, several lower molecular weight immunoreactive bands were detected as potential SBP2 degradation products or isoforms in wild-type HAP1 cells and not in ΔSBP2 cells, but the reason for protein degradation is unclear. Analysis of eEFSec localization in SBP2-null HAP1 cells demonstrated that presence or absence of SBP2 has no impact on the localization of eEFSec to the nucleus, suggesting that eEFSec and SBP2 are not imported into the nucleus as a complex. This result calls into question the idea that an active Sec incorporation mRNP forms in the nucleus as has been postulated as a mechanism by which a subset of selenoprotein mRNAs are protected from degradation by the NMD pathway [6]. It is impossible for us to rule out, of course, that SECISBP2L is responsible for some aspect of eEFSec localization in the absence of SBP2.

### eEFSec has a bipartite NLS

Proteins undergoing nucleocytoplasmic shuttling generally undergo facilitated diffusion across the nuclear pore and contain nuclear localization and export signals that allow them to interact with the transport receptors [28]. Using PSORTII, de Jesus et al identified an NLS in Domain IV of eEFSec and used mutagenesis to show that the predicted sequences indeed functioned as an NLS. Our findings using NLSMapper extend this analysis. NLSMapper, which scores each protein residue against all four classes of classical NLSs recognized by importin-α and is capable of identifying both monopartite and bipartite NLSs [18]. However, a monopartite NLS was not predicted. Instead, eEFSec was found to contain a bipartite NLS in Domain IV that included several novel residues predicted to participate in its nucleocytoplasmic shuttling in addition to those previously reported [6]. Bipartite NLSs are recognized by α-importin in a protein folding or conformation dependent manner, and these can sometimes be triggered by events such as phosphorylation [29,30]. Combined mutation of residues from both stretches of the predicted NLS resulted in loss of eEFSec signal in the nucleus, with deletion of eEFSec Domain IV resulting in almost complete nuclear exclusion. It is unclear what triggers the formation of an active bipartite NLS in eEFSec, although our data demonstrates that it is not cellular selenium status or the presence of SBP2.

### CRM1 is the likely eEFSec export receptor

The CRM1-mediated pathway is the major nuclear export pathway in eukaryotes. CRM1 (also known as exportin1) is a protein that interacts with nuclear pore complexes (NPCs) and also with leucine-rich NESs in a RanGTP dependent manner [31]. NES with regularly spaced hydrophobic residues other than Leu can also be recognized by CRM1, and therefore it has a wide range of substrates [23]. It can be reversibly inhibited by Leptomycin B (LMB), which covalently modifies a conserved cysteine residue in the substrate recognition region of CRM1 [32,33]. Components of the Sec incorporation machinery are also among CRM1 cargo: ribosomes have been shown to utilize CRM1 for nuclear export and in 2006, Papp et al showed that LMB treatment sequesters SBP2 in the nucleus over a 16 hour treatment duration, resulting in a two-fold reduction in selenoprotein synthesis. In this study, LMB treatment retained more eEFSec in the nucleus compared to untreated cells, whereas eEF1A was unresponsive, and a known LMB-responsive protein Cyclin B1 demonstrated efficient nuclear accumulation after as early 2 hours post-LMB treatment (reviewed in [23]). However, LMB is also cytotoxic and induces cell cycle arrest at G1 and G2 phases [34], so to eliminate any artifacts eEFSec distribution was also analyzed for cell-cycle dependent changes using synchronized cells and none were observed (data not shown). While our findings do not rule out that eEFSec is exported with ribosomes, we can conclude that eEFSec also utilizes CRM1-mediated nuclear export pathway.

### eEFSec Sec incorporation activity and nuclear localization are not coupled

In order to directly test whether nuclear localization of eEFSec is necessary for efficient selenoprotein production, eEFSec mutants would ideally be tested in an eEFSec-null cell line. Our efforts to generate eEFSec-null cells by the CRISPR/Cas9 gene editing system did not yield viable cell lines, consistent with genome-wide CRISPR/Cas9 knockout screen conducted in human cells that reported lethality in eEFSec-knockout cells [20]. Instead, we addressed the correlation between eEFSec localization and selenoprotein synthesis by analyzing subcellular distribution of eEFSec mutants whose Sec incorporation activity can be assessed in an in vitro assay, and by examining the impact of blocking eEFSec nuclear export on selenoprotein production.

Our lab has developed an eEFSec-dependent in vitro assay to test the effect of eEFSec mutations on Sec incorporation, and our previously published results show that mutation of LFKKE^480-484^ or KKRSR^536-540^ residues results in disruption of key aspects of eEFSec function, such as Sec-tRNA^Sec^ binding and SECIS/SBP2 binding, and inactivate the protein for Sec insertion [11]. LFKKE^480-484^ and KKRSR^536-540^ mutations did not reveal any localization defects, despite being critical to eEFSec activity. These findings also contrast previously published data which showed that mutating the two lysine residues in KKRSR to glutamic acid was sufficient to exclude eEFSec from the nucleus of HEK293 cells [6]. This difference can likely be attributed to differences in the nature of the mutations where the charge reversal of K→E is likely to result in more pronounced effects as compared to K→A mutation. However, it is also possible that the observed differences are due to inherent differences in eEFSec localization among various cell-types, the reason for which is not clear. Taken together, these findings highlight the fact that Domain IV mutations that disrupt eEFSec activity have no impact on its localization, suggesting that nuclear localization of eEFSec is not correlated with its participation in the Sec incorporation reaction, with the caveat that we are not able to reliably detect endogenous eEFSec, which could be playing a role in the observed localization of FLAG-eEFSec derived from the stably transfected plasmid.

Mutations in the conserved regions of Domains I, II and III show a variety of localization defects. Particularly interesting is the mutation of LARAL^23-27^, which results in complete nuclear exclusion of eEFSec. These residues are located about five residues upstream of eEFSec switch I region [17], and mutation of residues immediately 3’ of the switch II region has been previously shown to result in nuclear exclusion [6]. Switch regions of GTPases are known to be regions capable of conformational changes due to nucleotide binding or domain-domain interactions [35]. Due to the proximity of these residues to the switch I region of eEFSec, it is likely that their mutation alters the ability of eEFSec to undergo conformational changes due to either its inability to bind GTP or participate in inter-domain interactions. These findings implicate GTP binding to not only be important for a bipartite NLS, but also for inducing favorable conformation that is recognized by importin receptors. Therefore, Domain I, II and III mutants that are inactive and show disrupted shuttling are also likely to have an altered conformation, and potentially altered tRNA and GTP binding properties which may be necessary for nuclear localization. This is in contrast to the inactivating Domain IV mutations that should not result in Domain I-II-III conformational changes due to the fact that Domain IV is a distinct and distal structural element [17]. Further analysis of these mutants for tRNA binding, GTP hydrolysis and SBP2/SECIS binding will reveal more about the nature of their defects.

Altogether, the results presented here support the idea that a nuclear eEFSec does not participate in Sec incorporation complex, giving credence to a model where two separate pools of eEFSec exist in the nucleus and cytoplasm, and that the nuclear pools perform an as-yet unknown function. We can only speculate that the nuclear localization of eEFSec is the result of an intrinsic regulatory process that controls the amount that can accumulate in the cytoplasm, which may be important for maintaining the optimal stoichiometry with other Sec incorporation components.
